# Methylome evolution in plants

**DOI:** 10.1186/s13059-016-1127-5

**Published:** 2016-12-20

**Authors:** Amaryllis Vidalis, Daniel Živković, René Wardenaar, David Roquis, Aurélien Tellier, Frank Johannes

**Affiliations:** 1 Population Epigenetics and Epigenomics, Technical University of Munich, Liesel-Beckman-Str. 2, 85354 Freising, Germany; 2 Population Genetics, Technical University of Munich, Liesel-Beckman-Str. 2, 85354 Freising, Germany; 3 Groningen Bioinformatics Centre, University of Groningen, 9747 AG Groningen, The Netherlands; 4 Institute for Advanced Study, Technical University of Munich, Lichtenbergstr. 2a, 85748 Garching, Germany

## Abstract

Despite major progress in dissecting the molecular pathways that control DNA methylation patterns in plants, little is known about the mechanisms that shape plant methylomes over evolutionary time. Drawing on recent intra- and interspecific epigenomic studies, we show that methylome evolution over long timescales is largely a byproduct of genomic changes. By contrast, methylome evolution over short timescales appears to be driven mainly by spontaneous epimutational events. We argue that novel methods based on analyses of the methylation site frequency spectrum (mSFS) of natural populations can provide deeper insights into the evolutionary forces that act at each timescale.

**Electronic supplementary material:**

The online version of this article (doi:10.1186/s13059-016-1127-5) contains supplementary material, which is available to authorized users.

## Introduction

Cytosine methylation is a heritable epigenetic modification and a pervasive feature of most plant genomes [1–4]. It has important roles in regulating the expression of transposable elements (TEs), repeat sequences, and genes. Extensive experimental work has shown that changes in DNA methylation are associated with plant phenotypes [5–20], genome stability [21–25], polyploidization [26], recombination [27–31], and heterosis [32–40], and that such changes actively mediate environmental signaling [41–43], pathogen responses [44–46], and priming [47–49]. For these reasons, DNA methylation has emerged as a potentially important factor in plant evolution [50–53] and as a possible molecular target for the improvement of commercial crops [54, 55].

In the model plant *Arabidopsis thaliana*, cytosine methylation occurs in symmetrical CG and CHG contexts, as well as in asymmetrical CHH sequence contexts (where H = A, T, C) [56]. Extensive forward genetic screens in this species have made tremendous progress in dissecting the genetic pathways that establish and maintain context-specific methylation patterns throughout the genome [57]. These efforts have been facilitated by parallel technological developments in measuring methylomes at high resolution [58], which have permitted detailed assessments of the molecular impact of specific mutant genotypes.

Early methylome sequencing studies of the *A. thaliana* Columbia reference accession revealed that this model plant methylates about 10.5% of its cytosines globally (30% in context CG, 14% in CHG, and 6% in CHH, approximately), maintains dense methylation within TE and repeat sequences (at CG, CHG, and CHH sites), and (on average) intermediate methylation levels in gene bodies (mainly at CG sites) [59–62]. Insights into the evolutionary origin of these methylome features and into the forces that have shaped them over time cannot be readily obtained from experimental molecular studies, but require comprehensive inter- and intraspecific comparative epigenomic analyses. A major goal of these comparative approaches is to answer the following questions: ‘What are the factors that generate inter-individual variation in DNA methylation?’ and ‘How do evolutionary forces, such as selection, recombination and drift, act on this variation?’ A recent surge in fully sequenced plant genomes and methylomes is now providing the raw material that can be used to begin to answer these questions.

To date, the methylomes of about 90 diverse plant species have been analyzed by whole-genome bisulfite sequencing (WGBS-seq) [4, 57, 63–67] or by high-performance liquid chromatography (HPLC) [68]. These species include representatives of major taxonomic groups such as angiosperms (flowering plants), gymnosperms, ferns, and non-vascular plants, which diverged nearly 500 million years ago and thus cover much of the phylogenetic breadth of the plant kingdom. (For a list of plant species whose methylomes have been analyzed by WGBS-seq or by HPLC, and are analyzed in this Review see Additional file 1.) In addition to these interspecific data, deep genome and methylome sequencing has been performed for over 1000 natural *A. thaliana* accessions from all over the world [63, 69–75], as well as for several experimentally derived populations in *A. thaliana*, *Zea mays* and *Glycine max* [16, 17, 19, 76–80].

Here, we illustrate how these studies are beginning to provide deeper insights into methylome evolution in plants. Our review shows that long-term methylome evolution appears to be mainly a byproduct of genomic changes, such as the differential expansion of TE and repeat sequences as well as genetic mutations in pathways that control DNA methylation or transcriptional states. By contrast, short-term methylome evolution seems to be strongly dominated by heritable stochastic changes in DNA methylation (i.e., epimutations) that occur at relatively high rates and are largely independent of genomic backgrounds.

Because these two processes operate at different timescales, an obvious empirical goal is to be able to delineate their relative contributions to inter- and intraspecific methylome diversity patterns. We provide a proof-of-principle demonstration in *A. thaliana* showing that a formal analysis of the species’ methylation site frequency spectrum (mSFS) in terms of epimutational processes provides a powerful framework for addressing this challenge. We argue that further applications of such modeling approaches, in conjunction with high-throughput sequencing data, will be necessary to understand the forces that shape the evolution of plant methylomes over timescales that are of agricultural and evolutionary relevance.

## Methylome evolution over long timescales

Our understanding of the genome-wide properties of DNA methylation in plants has been deeply shaped by observations of *A. thaliana*, but this model plant of the *Brassicaceae* family has an unusually small and compact genome and a plastic methylome. Early comparisons between *A. thaliana* and several commercial crops, such as *Z. mays* and *Oryza sativa*, already signaled that many features of the *A. thaliana* methylome are not entirely representative of all plant species [64, 81–83]. In order to grasp the full evolutionary significance of these differences, and to be able to identify factors that can account for them, a more extensive phylogenic sampling of plant methylomes is necessary.

### Genome size and methylome diversity

**Fig. 1:**
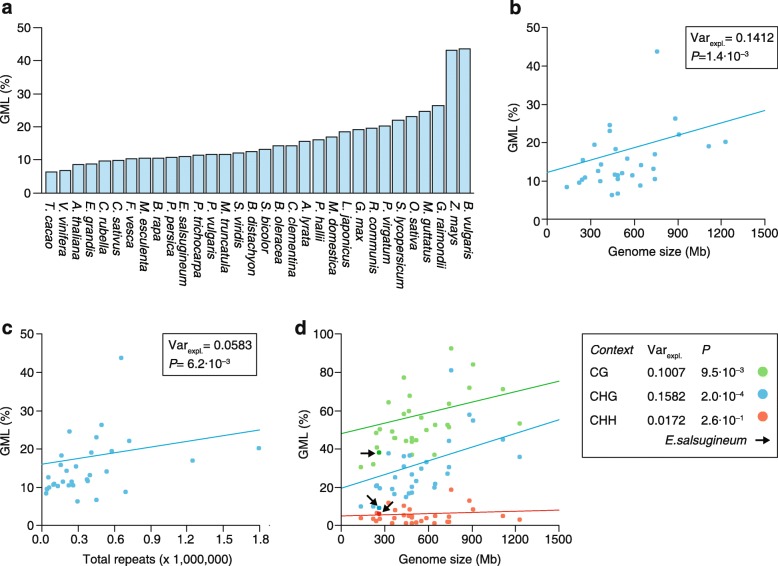
**a** Overview of genome-wide methylation levels (*GMLs*) in 32 angiosperm species as determined from whole-genome bisulfite sequencing data. GMLs approximate the percentage of all cytosines in the genome that are methylated. **b** Phylogeny-adjusted regression fit shows that genome size is positively correlated with GMLs, explaining about 14% of interspecific variation in GMLs (*Var*_*expl.*_). **c** Phylogeny-adjusted regression fit shows that the total number of annotated repeats is positively correlated with GMLs, explaining about 6% of interspecific variation in GMLs (*Var*_*expl.*_). **d** Phylogeny-adjusted regression fits show that genome size is correlated with context-specific GMLs in contexts CG and CHG, but not in context CHH. The *arrow* points to *Eutrema salsugineum*, a natural CMT3 mutant, which shows relatively low CHG- and CG-specific GMLs. Note: *Zea mays* was excluded from all regression analyses as it is an influential outlier because of its large genome size

Recent comparisons of 34 angiosperm methylomes show that genome-wide methylation levels (GMLs; a measure of the percentage of all cytosines that are methylated) can vary substantially between species even within the same taxon (Fig. 1a; see Additional file 2: Figure S1 for GMLs measured by HPLC and WGBS-seq). They range from as low as 5% in *Theobroma cacao* to as high as 43% in *Beta vulgaris*, with a mean of about 16% [3, 68]. While multiple factors probably contribute to these differences, interspecific variation in genome size is a strong predictor ([3, 68]; see Fig. 1b). Phylogeny-adjusted estimates show that about 14% of the diversity in GMLs is accounted for by variation in genome size (Fig. 1b). For every additional 100 Mbs of genomic sequence, GMLs increase by about 1.07%. This positive relationship can be explained by the fact that genome size differences are, to a large extent, the outcome of differential expansion of TEs and repeats [84, 85] (see Additional file 2: Figure S2), which are typically heavily methylated. Indeed, if the total number of annotated repeat copies in each species is used as a proxy for genome size, similar associations are detectable (Fig. 1c), although the effect sizes are somewhat smaller possibly owing to variation in repeat annotation quality [3].

These quantitative estimates support previous observations from a comparative analysis of three *Brassicaceae* species—*A. thaliana*, *Capsella rubella* and *Arabidopsis lyrata* [65]—which showed that methylome differences are mainly associated with centromeric expansion and deletion of repetitive sequences and TEs. In particular, the loss of three centromeres in *A. thaliana* relative to *A. lyrata* and *C. rubella* has led to a 10% reduction in its genome size and has a measurable impact on cytosine methylation distribution.

The extent of interspecific diversity in GMLs depends strongly on cytosine context. GMLs calculated from CG sites (i.e., CG-GMLs) vary only threefold between species, whereas for CHG-GMLs and CHH-GMLs, there is ninefold and 16-fold variation, respectively. Moreover, although genome size is associated with CG-GMLs and CHG-GMLs, there is no detectable association with CHH-GMLs (Fig. 1d). The biological source of these differences is not entirely clear, but may be at least in part due to technical difficulties in ascertaining CHH methylation states from WGBS-seq data in general [3, 4].

**Fig. 2:**
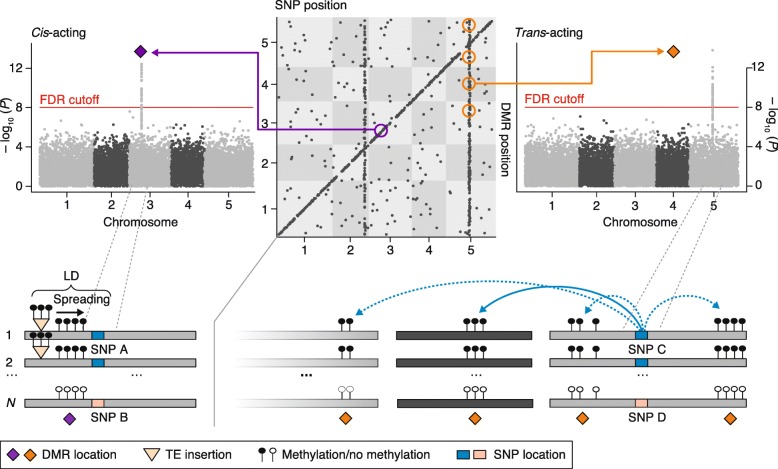
Schematic summary of a methylation quantitative trait locus (meQTL) mapping study in *A. thaliana* natural accessions. In the *cis-trans* plot (*top middle panel*), each dot represents a significant association between a single nucleotide polymorphism (*SNP*) and a differentially methylated region (*DMR*). All *cis* associations map along the diagonal, while *trans* associations are visible as vertical bands. An example of a commonly detected *cis* association is shown in the *left panel*. The SNP-DMR association is a byproduct of linkage disequilibrium (*LD*) between the SNP and an often undetected transposable element (*TE*) insertion that has spread methylation into its flanking region. An example of a commonly detected *trans*- association is shown in the *right panel*, where a SNP is associated with multiple DMRs across the genome. Such pleiotropic effects can be the result of SNPs in transcription factor genes or methyltransferase genes

Plant genome-size evolution can be relatively rapid [85, 86]. Even closely related local populations of *A. thaliana* natural accessions differ greatly in genome length [71]. Many of these differences appear to be driven by the differential expansion of 45S rDNA copies [71], which are typically targeted by DNA methylation [87]. Considerable copy-number differences in various TE families have also been documented among worldwide samples of *A. thaliana* [69, 88, 89]. Recent methylome analyses of these samples indicate that both old and new TE copies tend to be silenced by DNA methylation [88, 89], although de novo silencing of some classes of mobile copies may require several generations and depend on copy-number frequency [90]. It is well-known that, as a byproduct of TE and repeat silencing, DNA methylation often spreads from its target sites into flanking sequences [91, 92] and produces differentially methylated regions (DMRs) at the species level (Fig. 2). In the case of evolutionarily old insertions, such spreading-derived DMRs are effectively tagged by single nucleotide polymorphisms (SNPs) in linkage disequilibrium (LD) with the insertion sites (Fig. 2), and therefore appear as *cis* methylation quantitative trait loci (meQTL) in genome-wide association or linkage scans [63, 79, 93, 94]. Current estimates in *A. thaliana* and *Z. mays* suggest that about 20% and 50%, respectively, of all *cis*-meQTL are attributable to flanking structural variants [63, 94]. However, many TE insertions appear to have originated from very recent mobilization events and are therefore not associated with the underlying SNP haplotypes. Spreading of DNA methylation from such recent insertions produces rare epialleles that are not captured in meQTL studies, although they do contribute to methylome diversity at the species level [89].

### DNA methylation pathways and methylome diversity

Beyond genome-size evolution, inter- and intraspecific diversity in genome-wide and context-specific methylation levels can also be the outcome of genetic divergence in pathways that target DNA methylation. Studies with experimental mutants in *A. thaliana*, *Z. mays* and *O. sativa* show clearly that perturbations of de novo and maintenance methylation genes can strongly affect GMLs as well as context-specific methylation patterns [19, 95, 96]. Few natural experiments exist that would permit a comprehensive evaluation of the impact of such perturbations in the wild. Recently, Bewick et al. [97] reported that two angiosperm species, *Eutrema salsugineum* and *Conringia planisiliqua*, have independently lost CHROMOMETHYLASE 3 (CMT3), an essential methyltransferase that catalyzes histone H3 lysine 9 di-methylation (H3K9me2)-associated CHG methylation [98]. These natural mutants show significantly reduced gene body methylation as well as a reduction in global CHG methylation levels ([3, 97]; Fig. 1d).

Even single point mutations in otherwise highly homologous genes are sufficient to generate extensive methylation diversity. Dubin et al. [73], for instance, used a meQTL mapping approach to show that two *trans*-acting SNPs in the gene encoding CHROMOMETHYLASE 2 (a homologue of CMT3) substantially alter CHH methylation levels among *A. thaliana* accessions sampled from the north and south of Sweden, and another causative polymorphism in this gene has been identified in larger Eurasian samples [99]. Furthermore, Quadrana et al. [88] recently performed a genome-wide association (GWA) analysis in *A. thaliana* accessions in which they treated TE copy number as a quantitative trait. Their scan identified a candidate causal SNP in the gene encoding MET2a, a poorly characterized homologue of the CG methyltransferase MET1 [100, 101]. This example illustrates that genetic mutations that affect DNA methylation pathways can act as facilitators of genomic changes, and set into motion complex co-evolutionary dynamics between genomes and epigenomes.

The systematic identification of similar pathway mutations is far more challenging in the context of interspecific analysis. Many genes are involved in DNA methylation pathways [56, 102], and so a comprehensive investigation of gene family phylogenies would be necessary to reveal connections between the functional conservation of specific orthologs and methylome diversity patterns. To date, such information is on the way for the *CMT* gene family [103], but only limited results are currently available for other methyltransferase genes or other DNA methylation-related genes [1, 4, 102, 104]. Potential insights from such an analysis are further complicated by the fact that the functional consequences of pathway mutations can be highly dependent on genomic backgrounds. This is exemplified by failed attempts to construct composite loss-of-function mutations in DNA methylation genes in *Z. mays* [19], even though similar mutations are fully viable in *A. thaliana* [95].

Nonetheless, Niederhuth et al. [3] recently argued that an indirect approach to assessing the differential efficiency of DNA methylation pathways across species is to compare measures of intragenomic variability in site-specific methylation levels or in the degree of strand-symmetrical methylation at CG and CHG sites. In this formulation, a methylation pathway is considered robust if intragenomic variability is low and symmetrical methylation at CG and CHG is high. The fact that angiosperms differ substantially along these metrics suggests that methylation maintenance efficiency is species-dependent, even if the underlying pathway perturbations remain unknown. These metrics are certainly interesting but need to be evaluated carefully with regards to technical confounders such as mappability, genome coverage, and differential heterogeneity of the sampled tissues.

### Gene-body methylation (gbM) as a neutral byproduct of transcription

Arguably one of the most enigmatic features of plant methylomes is the methylation of gene bodies. Body methylated (BM) genes have been heuristically defined as genes that methylate more than 90% of their CG sites and less than 5% of their CHG and CHH sites [105]. The latter requirement filters out genes that feature TE-like methylation patterns, perhaps because they were originally derived from TEs or contain intact or degenerate TE copies. In *A. thaliana*, about 18% of genes are BM whereas about 65% are unmethylated (UM). Unlike its repressive role in TEs and repeats, methylation in gene bodies tends to occur in moderate to highly expressed genes [62, 97]. The molecular mechanisms by which gene-body methylation (gbM) contributes to transcription, if at all, and its evolutionary significance are not fully understood.

### gbM is associated with evolutionarily important genes

Indirect evidence that gbM may be evolutionarily important has come from the observation that BM and UM genes in *A. thaliana* differ markedly in sequence composition. BM genes are about twofold longer and have greater exon content [105]. Moreover, comparisons of *A. thaliana* and *A. lyrata* orthologs show that the ratio of nonsynonymous (*K*_*A*_) to synonymous (*K*_*S*_) substitutions is markedly lower in BM than in UM genes (*K*_*A*_/*K*_*S*_ = 0.198 and *K*_*A*_/*K*_*S*_ = 0.23, respectively; *p* < 10^−5^), suggesting that BM genes are subject to stronger evolutionary constraints. Interestingly, in addition to the lower *K*_*A*_/*K*_*S*_ ratio, BM genes seem to evolve at a slower rate in general. This is evidenced by the fact that the actual rate of, presumably neutral, synonymous (*K*_*S*_) and intron (*K*_*INT*_) divergence is significantly reduced in BM compared with UM genes (*K*_*S*_ = 0.122 in BM and 0.140 in UM, *K*_*INT*_ = 0.107 in BM and 0.137 in UM). In support of this argument, Takuno and Gaut [105] showed that nucleosome occupancy is positively correlated with *K*_*S*_ and *K*_*INT*_ values, attributing this to more efficient DNA-repair machinery in nucleosome-free regions [105]. However, the DNA-repair argument does not readily extend to CG dinucleotides: BM genes are highly depleted in GC content as well as in the proportion of CpG dinucleotides compared with UM genes, which reflects the well-known mutagenic potential of methylated cytosine to change to thymine as a result of deamination [105]. That this difference in CG content is so visible in current sequencing data suggests that methylation levels in gene bodies must have been maintained for significant evolutionary periods.

#### The selection hypothesis

But how can gbM be maintained as evolution proceeds while methylated cytosines are continually lost through deamination? One explanation for this paradox is that gbM, itself, is under positive selection, which would result in an equilibrium CG content that is defined by the balance between the rate of deamination and the strength of selection [106, 107]. This selection hypothesis implicitly assumes that gbM is essential for gene function, and should therefore be conserved between orthologs across plant species. Initial methylome comparisons between two related grasses, *Brachypodium distachyon* and *O. sativa*, seemed to support this prediction [106], but more extensive taxonomic sampling now shows that gbM can be highly variable across species [4], even within the same taxonomic groups [3, 97]. The most extreme cases are the two angiosperm species that have no CMT3 (*E. salsugineum* and *C. planisiliqua*) and lack gbM altogether. Despite the loss of gbM, the transcriptional state of orthologous genes in these two species seems to be largely conserved, suggesting that gbM has no causal role in the functional conservation of these orthologs.

#### The emerging neutrality hypothesis

The potential uncoupling of gbM from transcriptional states has raised the question of why gbM often appears in moderately and highly expressed genes in the first place. An emerging hypothesis is that gbM is simply the neutral byproduct of active transcription. Bewick et al. [97] recently proposed a molecular model for this neutrality hypothesis in which H3K9me2 is stochastically incorporated into transcribed genes. The transient presence of H3K9me2 kickstarts CMT3-dependent methylation of CHG sites and occasionally leads to the methylation of neighboring CGs, which are then maintained by the CG methyltransferase MET1. However, not all transcribed genes are body methylated. Bewick et al. [97] identified additional sequence and chromatin features that facilitate the accumulation of gbM within transcribed genes.

The hypothesis that gbM is a neutral byproduct of transcription predicts that it should, at least partly, track the evolution of transcriptional states in plant populations, provided that the full DNA methylation machinery is in place. Preliminary evidence that supports this prediction comes from a recent integrative transcriptome and methylome analysis in *A. thaliana* natural accessions [108]. Causal modeling shows that most *cis*- or *trans*-acting SNPs that are associated with both the expression and the methylation levels of a given gene tend to affect methylation through their effects on gene expression rather than the other way around. In other words, methylation is a byproduct of genetic effects on transcription. Many of these causal SNPs show evidence of positive selection [73], suggesting that the evolving genetic basis that underlies these transcriptional states leaves secondary signatures at the level of gbM.

## Methylome evolution over short timescales

**Fig. 3:**
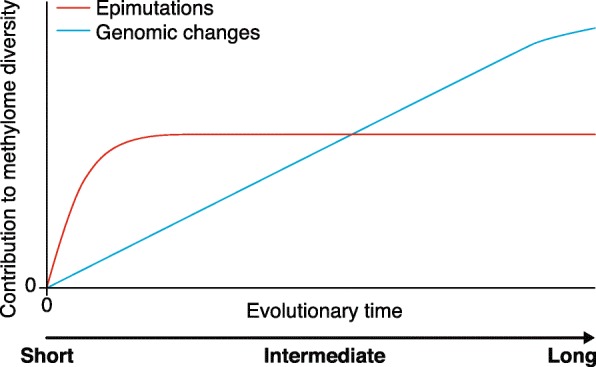
We propose a heuristic model whereby genomic changes and spontaneous epimutations occur simultaneously, and contribute to interspecific or intraspecific methylome diversity over evolutionary time. For illustrative purposes, we assume that lineages descended from a common founder plant at time 0. The rapid accumulation of heritable epimutations dominates methylome diversity over short timescales but quickly converges to an equilibrium diversity value that is defined by the magnitude of forward and backward epimutation rates as well as by their ratios (i.e., the epimutation bias parameter). By contrast, genomic changes accumulate more gradually among lineages, and begin to dominate methylome diversity after longer evolutionary divergence times. An important empirical challenge is to delineate the relative contributions of these two processes based on methylome diversity data collected at any point along this evolutionary trajectory. Recent theoretical models for the analysis of the methylation site frequency spectrum (mSFS) provide an important step in this direction

As discussed above, our current state of knowledge points to genomic changes as a major cause of long-term methylome evolution. These genomic changes can be in the form of repeat or TE expansion or the result of genetic perturbations in pathways that control DNA methylation or transcriptional states. The species-level methylome footprints of these changes are expected to emerge gradually, as point or structural mutations need to arise first and then spread within or among populations through selection and drift (Fig. 3). An intriguing observation, however, is that heritable alterations in DNA methylation states can also emerge spontaneously and independently of genetic mutations [8, 57, 76–78, 109–113]. The most comprehensive demonstration of this phenomenon has come from the analysis of multi-generational methylome data from *A. thaliana* mutation accumulation lines (MA-lines) [76–78, 112]. Such lines are derived from a single founder plant (of the Columbia accession) and independently propagated for a large number of generations [114]. Detailed comparisons of the methylomes of MA-lines have been instrumental in providing the first estimates of the rate at which epimutations occur in plant genomes [76–78]. Efforts are now underway to try to understand the extent to which spontaneous epimutations contribute to methylome diversity in natural populations over short timescales up to thousands of generations.

### Spontaneous epimutations can rapidly generate methylome diversity

Spontaneous epimutations can be defined as heritable stochastic changes in the methylation status of individual cytosines or of clusters of cytosines. In plants, such stochastic events can occur at CG, CHG, and CHH sites. The meiotic inheritance of epimutations, however, appears to be mainly restricted to CG dinucleotides [76–78], probably as a result of context-specific methylation resetting during gametogenesis and early development [115]. Estimates in MA-lines indicate that the rate of forward epimutations (i.e., stochastic gains of methylation) is about 2.56 × 10^−4^ per CG site per haploid genome per generation, while the rate of backward epimutations (i.e., stochastic loss of methylation) is about 6.3 × 10^−4^ [78]. Hence, methylation loss is globally about 2.5 times as likely as methylation gain. The asymmetry in these rates has immediate consequences for understanding GMLs in *A. thaliana*: it implies that about 30% of all CG dinucleotides should be methylated at equilibrium and 70% unmethylated, provided that evolutionary forces such as selection and gene conversion are negligible. These percentages are roughly consistent with actual measurements of GMLs in the *A. thaliana* reference accession (Columbia), suggesting that epimutations are fundamental to methylome evolution despite the myriad of ways in which genomic changes can shape methylation patterns in the long term.

Putting these rates into perspective, the rate of CG epimutations is about five orders of magnitude higher than the rate of genetic mutations in *A. thaliana* (7 × 10^−9^) [116]. In sheer numbers, about 10,000 CG methylation changes occur in a single generation, which contrasts with the two base changes resulting from genetic mutations. The fast accumulation of these methylation changes causes methylomes to diverge rapidly over short timescales. Even after only 30 generations of independent selfing, the methylomes of early-generation and late-generation MA-lines can be clearly distinguished. As the methylation status of individual CG sites is simultaneously subject to both forward and backward epimutations, methylome divergence does not increase linearly over time [72, 78, 117] but saturates rather quickly to some equilibrium divergence value (Fig. 3). On the basis of estimates from Van der Graaf et al. [78], only about 4000 generations would be needed in a hypothetical mutation accumulation experiment for methylome divergence to be within 99% of this value. This insight leads to the evolutionary prediction that epimutational processes should dominate methylome diversity in the early stages of lineage divergence but only marginally at later stages.

The high epimutation rates have additional implications for studying methylome diversity within and across populations. First, the observed shared methylated state between two individuals (so-called identity by state) cannot be assumed automatically to be inherited from the same parent (so-called identity by descent), because it could have been generated by independent epimutation events. This concept is defined as homoplasy and has been largely studied for microsatellite markers [118]. Second, as divergence in the methylome between populations increases rapidly, backward and forward epimutations would occur at many sites. Therefore, homoplasy will be observed when comparing diverged populations of the same species, thus decreasing the accuracy of inference of past evolutionary events.

### Epimutation-induced methylome diversity patterns are potentially long-lived

**Fig. 4:**
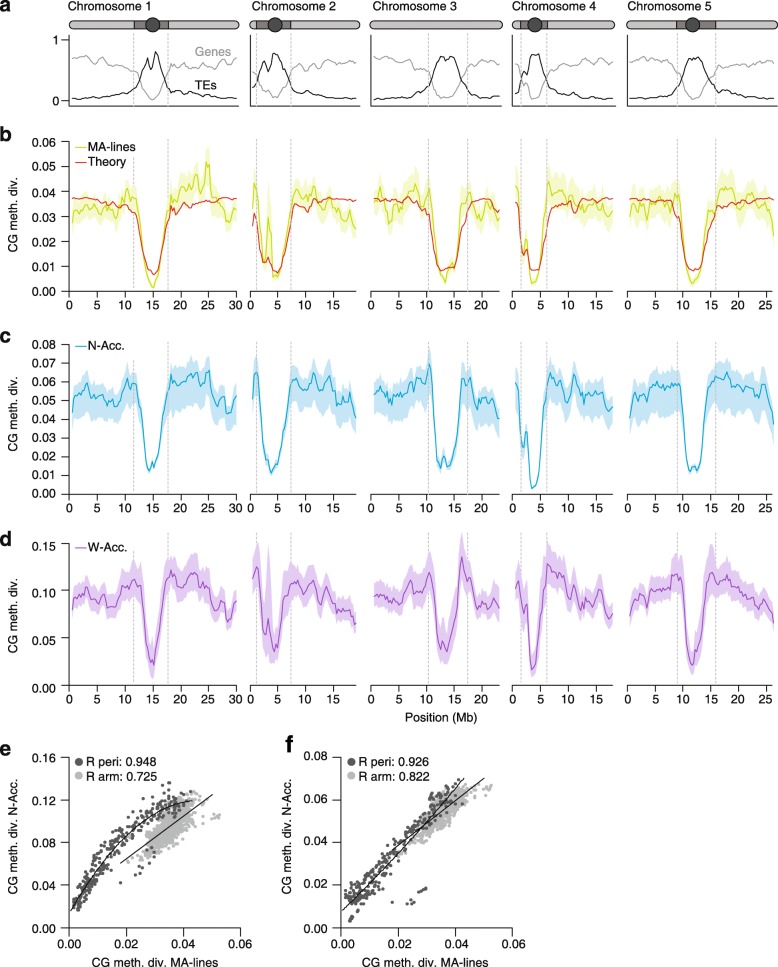
**a** Gene (*light gray*) and transposable element (*TE*) (*dark gray*) densities along the *A. thaliana* genome (Columbia reference). A schematic representation of the five chromosomes is shown above (*circle*, centromere; *dark gray*, pericentromeric region; *light gray*, arm). **b** Annotation-specific CG epimutations produce distinct methylome diversity (*CG meth. div.*) patterns among mutation accumulation lines (*MA-lines*) that have diverged for merely 30 generations (average diversity was calculated in 1 Mb sliding windows, step size 100 kb). These diversity patterns can be predicted from annotation-specific estimates of epimutation rate and the density distribution of annotation units along the genome (*red theoretical line*). **c** CG methylome diversity (*CG meth. div.*) patterns among 13 North American accessions (*N-Acc.*) (after around 200 generations of divergence). **d** Methylome diversity patterns among 138 worldwide accessions (*W-Acc*.) (after several hundred thousand years of divergence). **e** CG methylome diversity patterns are significantly correlated between the MA-lines and the W-Acc., both in pericentromeric (*peri*) regions (*dark gray dots*) as well as in euchromatic chromosome arms (*light gray dots*). **f** These correlations are even stronger when MA-lines are compared to the N-Acc., suggesting that the accumulation of DNA sequence polymorphism has perturbed epimutation-induced methylome diversity patterns over time

Like genetic mutations, CG epimutations are not uniformly distributed across the genome, but vary in rate between different annotation contexts [76–78, 112]. In *A. thaliana*, the highest combined forward and backward rates are found in genes, with the forward rate (3.48 × 10^−4^) being about four times lower than the backward rate (1.47 × 10^−3^). In TEs, by contrast, these rates are much reduced, and the forward rate (3.24 × 10^−4^) exceeds the backward rate (1.20 × 10^−5^) by a factor of 30 [78]. The strong epimutation bias toward methylation gain in TEs is consistent with constitutive silencing of these sequences by DNA methylation. An important by-product of these annotation-specific rates (and their degree of asymmetry) is that some genomic regions diverge faster than others and also tend toward distinct equilibrium divergence values over time. That is, CG epimutations are expected to produce methylome diversity patterns along chromosomes that closely reflect the spatial distribution of various annotation units (i.e., chromosome architecture) (Fig. 4). In the *A. thaliana* MA-lines, this can be seen clearly when comparing pericentromeric regions (TE-rich) and chromosome arms (gene-rich), with the latter being on average about 2.3 times more divergent than the former (Fig. 4).

Because chromosome architecture is broadly stable over long evolutionary timescales, the signatures of epimutational events are potentially long-lived. Indeed, a striking observation is that the epimutation-induced methylome diversity patterns in the MA-lines are highly correlated with those seen among worldwide natural accessions (pericentromeric regions: *ρ* = 0.94, chromosome arms: *ρ* = 0.72; Fig. 4), despite the latter having diverged for hundreds of thousands of years [119, 120]. These correlations are even stronger, particularly in chromosome arms, when the MA-lines are compared to a selected sample of North American natural accessions that diverged from a common founder about 200 years ago [72] (pericentromeric regions: *ρ* = 0.92, chromosome arms: *ρ* = 0.82; Fig. 4). Together, these observations indicate that—while the accumulation of sequence polymorphisms affects methylation diversity patterns over time—in the current state of the species’ evolutionary trajectory, these effects are not overwhelming. Similar conclusions can be reached on the basis of a careful evaluation of meQTL studies in *A. thaliana* accessions [63, 73, 75], which show that on average only about 18–35% of all DMRs are associated with *cis*- or *trans*-acting sequence polymorphisms [93]. The above insights raise the following important questions. Are spontaneous epimutations generally a major cause of methylome diversity in natural plant populations? And if so, what are the evolutionary forces that act on these epimutations?

## Analysis of the methylation site frequency spectrum (mSFS)

**Fig. 5:**
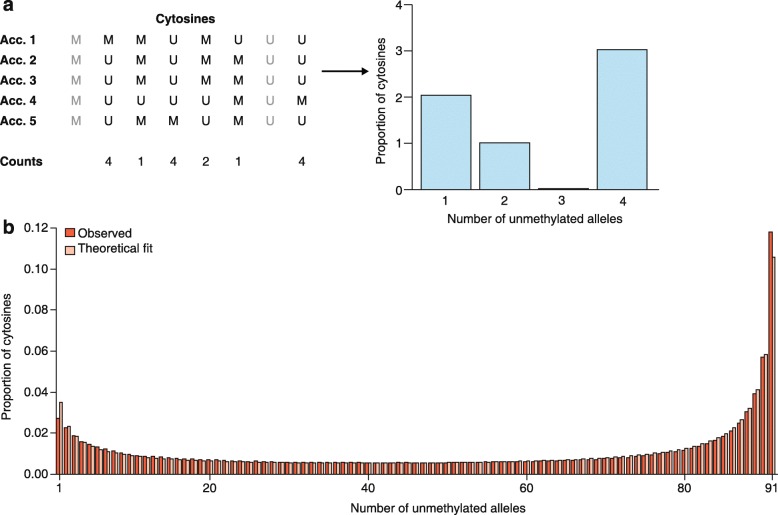
**a** Simplification of the reconstruction of a methylation site frequency spectrum (mSFS). In this example, we consider a sample size of five accessions (*Acc.*), and eight sites among which two (in *gray*) are monomorphic and thus discarded for the mSFS. For each cytosine, each accession might exhibit a methylated (*M*) or an unmethylated (*U*) state. For the mSFS, counts are taken of the number of accessions that are unmethylated for that cytosine. These counts define discrete epiallelic classes (number of unmethylated alleles). **b** The observed frequencies of each epiallelic class is determined, in this case, from genic CG sites of 92 *A. thaliana* worldwide natural accessions (*red bars*), along with the maximum likelihood estimate based on the theoretical result of Charlesworth and Jain [123] (*pink bars*). The theoretical model (see Box 1) provides an accurate fit to the observed genic CG methylation diversity patterns, suggesting that CG epimutations are a major factor in shaping methylome diversity in natural populations of *A. thaliana* over evolutionary timescales

One way to approach these questions is to analyze the mSFS (Fig. 5) using a theoretical model that explicitly accounts for forward and backward epimutations as well as for evolutionary forces such as selection and drift. Although this modeling approach goes back to Wright [121], results that are applicable for the analysis of genomic data have been obtained recently [122–124]. More popularized classic population genetics models that assume irreversible mutations (see also Wright [121]) on infinitely many sites [125], as is often the case for genomic data, are not suitable in the context of epimutations because of their reversibility and relatively high asymmetric rates. Recently, Charlesworth and Jain [123] derived analytical results based on the work of Wright [121], which incorporate many of the key features of epimutations (Box 1). Their formulas can be directly applied to WGBS-seq data that describe single methylation polymorphisms (SMPs) or DMRs to make inferences about the evolutionary role of epimutations and selection in shaping methylome diversity patterns in natural populations.

### Analysis of mSFS in *A. thaliana*: an example

To demonstrate the power of this approach, we constructed the mSFS from public WGBS-seq data of 92 worldwide natural *A. thaliana* accessions [63] (Fig. 5; see Additional file 3 for a description of how the methylomes used for the mSFS calculations were filtered). These 92 accessions represent a so-called species-wide sample of *A. thaliana*, characterizing the collecting phase of the species’ coalescent tree [126]. This sample can be seen as a panmictic population and thus fulfills our model’s assumptions (Box 1). For this analysis, we focused only on genic CG sites, because this approach allowed us to draw connections between epimutational processes and the nature of gbM discussed above. As shown in Fig. 5, our theory-based estimates give an accurate description of the observed mSFS, indicating that the underlying model assumptions are sufficient and that epimutations are a major factor in shaping species-level methylome diversity in *A. thaliana*. Several important insights are emerging from this model fit.

First, the best fitting model provides no evidence for selection on genic CG epimutations at the genome-wide level. This observation is consistent with earlier theoretical models of the MA-lines, which have shown that epimutations accumulate neutrally under benign environmental conditions and in nearly isogenic sequence backgrounds [78]. The lack of selection also provides support to the molecular model of Bewick et al. [97], which posits that gbM is essentially a neutral by-product of expression, although a more detailed mSFS analysis that considers separate classes of BM and UM genes will be required to confirm this.

A second major insight from the mSFS fit is that the ratio of forward and backward population epimutation rates is similar to that estimated in the MA-lines (3.43 vs. 4.24, respectively). This result indicates that the epimutation bias parameter is robustly maintained in natural environments and in the context of varying genomic backgrounds, a conclusion that has also been reached by Hagmann et al. [72] using less formal arguments. Estimates of the actual epimutation rates, however, are not available from the mSFS output because the population epimutation parameters are a function of the effective population size (*N*_*e*_), and cannot be disentangled (Box 1). This is unfortunate as it would be interesting to assess the extent to which the actual rates are modulated by environmental and genetic factors. A previous experiment in which MA-lines were derived under high-salinity soil conditions provided evidence that epimutations are more frequent under this stressor [112]. Similar experiments are underway to assess the rate and spectrum of epimutations as a function of varying genomic backgrounds.

### Interesting future directions in the analysis of mSFS

The mSFS analysis approach opens up exciting research avenues. Most notably, it provides a formal framework for carrying out methylome-wide scans for signatures of epigenetic selection by identifying DMRs that significantly diverge from the expected mSFS. While the interpretation of such regions is difficult, as they could be the result of direct selection on methylation states or the outcome of indirect selection on *cis*- or *trans*-acting genetic variants, this approach would provide a way to prioritize regions for further analytical or experimental analysis. These methylome-wide scans will also provide a new perspective on the large number of methylomes that have been recently collected in *A. thaliana*, or will be collected for other plant species in the near future. Another interesting extension of the mSFS approach is to generalize the theoretical result of Charlesworth and Jain [123] to account for time dependence and therefore to incorporate changes in the population size. Such a model could be used in conjunction with genic CG mSFS data to define a kind of ‘fast-ticking’ molecular clock. Genic CG epimutations can be considered as neutral and occur at rates far exceeding the genetic mutation rate, and so such a re-calibrated clock would yield high-resolution insights into very recent demographic events that would otherwise be invisible on the basis of DNA sequencing data alone.

## Conclusions

The recent availability of high-resolution inter- and intraspecific methylome data is providing new insights into the evolutionary role of DNA methylation in plants. Such insights complement the tremendous progress made in recent years in understanding more proximal questions regarding the molecular mechanisms that control DNA methylation during the life course of a plant and during its reproductive stages. This review provides a first unified framework for understanding the evolution of methylation in plants, based on the fact that the epigenomic divergence observed at the longer timescales is necessarily the result of processes occurring within populations at shorter timescales.

At the population level, spontaneous epimutations appear to be a major factor in generating methylome diversity. These epimutations are characterized by their high, asymmetric rates, and the fact that they occur at a finite number of cytosines. Following population genetics theory, drift and selection should drive the changes in epimutation frequencies over time, thus shaping the mSFS in a population. We predict that most plant populations will be close to statistical equilibrium with respect to epimutation, genetic drift, and selection, and that they will be characterized by extensive homoplasy. Cases of positive or purifying selection on epialleles have never been reported, probably because of a lack of appropriate statistical analyses. Hence, an open question is whether epigenetic selection is pervasive or rare in plant populations. A theory-based analysis of the empirical mSFS provides a framework for detecting signatures of positive and purifying selection at the genome-wide scale. Using such an approach, future studies should assess the extent to which the mSFS for different annotation units is conserved between plant species. For instance, is the neutral mSFS that we have detected in *A. thaliana* natural populations typical? The fact that genic sequences in complex genomes are often ‘contaminated’ with TEs and sequence repeats [4] would suggest that epimutation dynamics differ fundamentally among different genomes and may be subject to selection. Population-level methylome data in several other plant species will soon emerge to answer these questions.

When populations diverge, drift and high epimutation rates generate fast divergence in methylation at existing cytosine sites. If local adaptation occurs and is mediated by DNA methylation, selection should be observable in the mSFS, and possibly also with the greater divergence between populations of mSFS in key genes for adaptation. Within populations, more drastic genomic changes will arise slowly; these might include, for example, genome rearrangements, gene duplication, the repeating or expansion of TEs, changes in methylation pathways, and so on. We know that these genomic changes affect methylation patterns because DMRs are often associated with segregating structural variants or with mutations in methyltransferase genes. When these features become fixed in a population, the methylome landscape changes drastically. This can be then observed in comparative epigenomics studies that show the cumulative outcome of genetic changes.

From a theoretical perspective, a crucial future step is to develop models that bridge these different time and spatial scales. Such models should include not only population genetic processes (drift, epimutation, recombination, migration, and selection) but also genomic rearrangements and TE dynamics to derive testable hypotheses and statistics suited for the analysis of intra- and interpopulation and species data.

These data-driven modeling efforts should be complemented by rigorous experimental studies that determine how heritable DNA methylation changes arise in different plant species and mating systems, and the extent to which these changes contribute to plant fitness and respond to artificial or natural selection.

## Box 1

### Analysis of the methylation site frequency spectrum (mSFS)

Consider a randomly mating, panmictic, diploid population with constant population size N. Each cytosine has two epiallelic states $cM$ and $cU$, with the former denoting a methylated and the latter an unmethylated state. We assume that forward epimutations ($cU$→$cM$ *)* occur at rate *α* = 4*Nμ*_*UM*_, and backward epimutations ($cM$→$cU$) at rate *β* = 4*Nμ*_*MU*_. Selection acts with coefficient *σ* = 2*Ns*, where the relative fitness of the $cU$/$cU$ and $cM$/$cU$ epigenotypes over $cM$/$cM$ are given by 1 + 2$s$ and 1+$s$, respectively. According to Charlesworth and Jain [123] the probability that a sample of size n segregates for b $cU$ variants (with 0 ≤ $b$ ≤ $n$) is

$$ {p}_{n,b}=\left(\frac{n}{b}\right)\frac{F\left(\beta +b;a+\beta +n;2\sigma \right){\beta}_{(b)}{\alpha}_{\left(n-b\right)}}{F\left(\beta, \alpha +\beta, 2\sigma \right){\left(\alpha +\beta \right)}_{(n)}} $$

Where *F* (*x*;*y*;*z*) denotes the confluent hypergeometric function of the first kind and the *d*_*(j)*_ are rising factorials [127]. Note that the equation has been slightly adapted to our notation. The proportion of segregating sites is *p*_*seg*_ = 1-*p*(*0*)-*p*(*n*) and the mSFS is obtained as

$$ {q}_{n,b}={p}_{n,b}/{p}_{seg} $$

We introduce this equation into a maximum likelihood framework to infer the epimutation rates and the selection coefficient from the observed mSFS, which can be constructed from whole genome bisulphite sequencing data. Assuming independent sites, the log-likelihood of a model $M$ given data $D$ is

$$ \log \left[L\left(D;M\right)\right]={\displaystyle \sum_{b=1}^{n-1}{d}_{n,b}} \log \left({q}_{n,b}\right)+ constant, $$

Where *d*_*n,b*_ is the observed number of sites at which the $cU$ epiallele occurs $b$ times in the sample, and *q*_*n,b*_ is the probability that the $cU$ epiallele occurs *b* times in the sample at a segregating site under model *M* [128]. To emphasize the proportion of the two epimutation rates α and β, we use the epimutation bias parameter r via β = r$\alpha$. Maximum likelihood estimates for the parameters r, $\alpha$ (thus β) and σ can be obtained by performing a grid search over the parameter space. The model with the highest likelihood is selected.

Note that the mSFS approach is also applicable when using ‘regions’ (i.e. clusters of cytosines) as units of analysis rather than individual cytosines. However, this shift in focus requires that differentially methylated regions (DMRs) can be assumed to exist in two epialleic states (i.e. methylated and unmethylated) and that epimutation events occur at the level of ‘regions’.

## Additional files

Additional file 1: Plant species whose methylomes have been analyzed by whole-genome bisulfite sequencing (WGBS-seq) or by high-performance liquid chromatography (HPLC). (PDF 277 kb)
Additional file 2: Figure S1. GMLs of different taxa measured by HPLC and WGBS-seq. **Figure S2.** Correlation between genome size and total number of repeats in the genome. (PDF 1044 kb)
Additional file 3: Filtering of the methylomes used for the calculation of the mSFS. (DOCX 17 kb)

## Abbreviations

BM: Body methylated
CMT3: CHROMOMETHYLASE 3
DMR: Differentially methylated region
gbM: Gene-body methylation
GML: Genome-wide methylation level
H3K9me2: Histone H3 lysine 9 di-methylation
HPLC: High-performance liquid chromatography
*K*_*A*_: Number of non-synonymous substitutions per non-synonymous sites
*K*_*INT*_: Intron divergence
*K*_*S*_: Number of synonymous substitutions per synonymous sites
LD: Linkage disequilibrium
MA-line: Mutation accumulation line
meQTL: Methylation quantitative trait loci
mSFS: Methylation site frequency spectrum
SNP: Single nucleotide polymorphism
TE: Transposable element
UM: Unmethylated
WGBS-seq: Whole-genome bisulfite sequencing
