# Erratum to: Methylome Evolution in plants

**DOI:** 10.1186/s13059-017-1176-4

**Published:** 2017-02-27

**Authors:** Amaryllis Vidalis, Daniel Živković, René Wardenaar, David Roquis, Aurélien Tellier, Frank Johannes

**Affiliations:** 1 Population Epigenetics and Epigenomics, Technical University of Munich, Liesel-Beckman-Str. 2, 85354 Freising, Germany; 2 Population Genetics, Technical University of Munich, Liesel-Beckman-Str. 2, 85354 Freising, Germany; 3 Groningen Bioinformatics Centre, University of Groningen, 9747 AG Groningen, The Netherlands; 4 Institute for Advanced Study, Technical University of Munich, Lichtenbergstr. 2a, 85748 Garching, Germany

## Erratum

After publication of this article [1] we noticed that the centromere of Chromosome 3 was missing from Fig. 4a, and that the Fig. 4e y-axis should read ‘CG meth. Div. W-Acc.’. The y-axis of the barplot in Fig. 5a should read ‘Number of cytosines’. The corrected Figs. 4 and 5 are shown below.

**Fig. 4:**
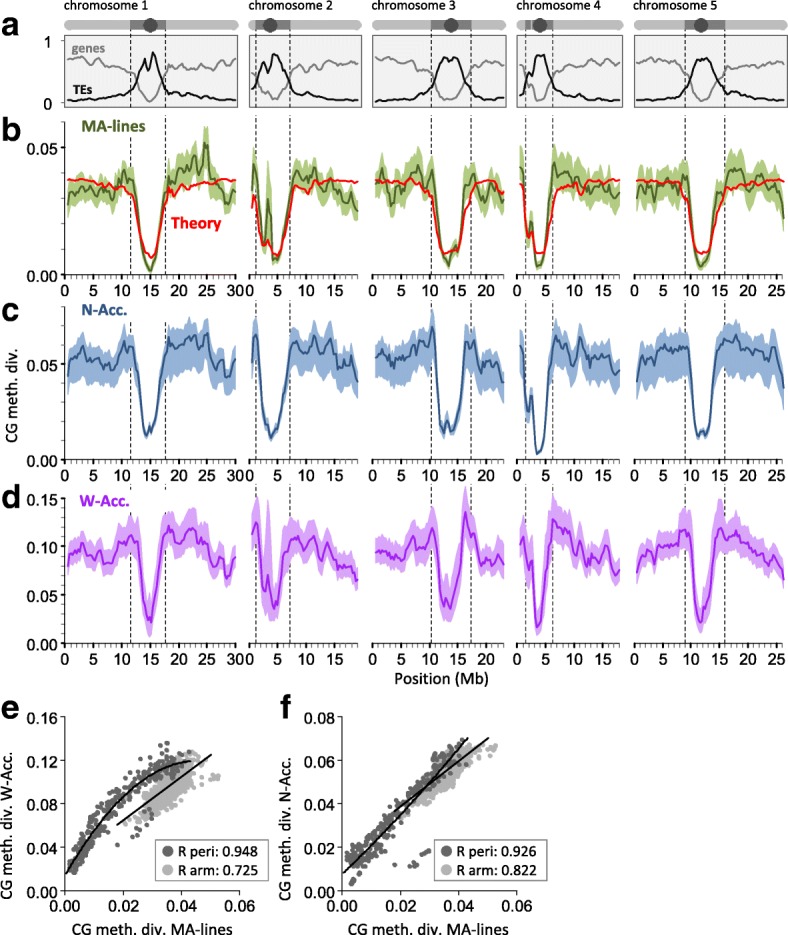
**a** Gene (*light gray*) and transposable element (TE) (*dark gray*) densities along the A. thaliana genome (Columbia reference). A schematic representation of the five chromosomes is shown above (circle, centromere; *dark gray*, pericentromeric region; *light gray*, arm). **b** Annotation-specific CG epimutations produce distinct methylome diversity (CG meth. div.) patterns among mutation accumulation lines (MA-lines) that have diverged for merely 30 generations (average diversity was calculated in 1 Mb sliding windows, step size 100 kb). These diversity patterns can be predicted from annotation-specific estimates of epimutation rate and the density distribution of annotation units along the genome (*red theoretical line*). **c** CG methylome diversity (CG meth. div.) patterns among 13 North American accessions (N-Acc.) (after around 200 generations of divergence). **d** Methylome diversity patterns among 138 worldwide accessions (W-Acc.) (after several hundred thousand years of divergence). **e** CG methylome diversity patterns are significantly correlated between the MA-lines and the W-Acc., both in pericentromeric (peri) regions (*dark gray dots*) as well as in euchromatic chromosome arms (*light gray dots*). **f** These correlations are even stronger when MA-lines are compared to the N-Acc., suggesting that the accumulation of DNA sequence polymorphism has perturbed epimutation-induced methylome diversity patterns over time

**Fig. 5:**
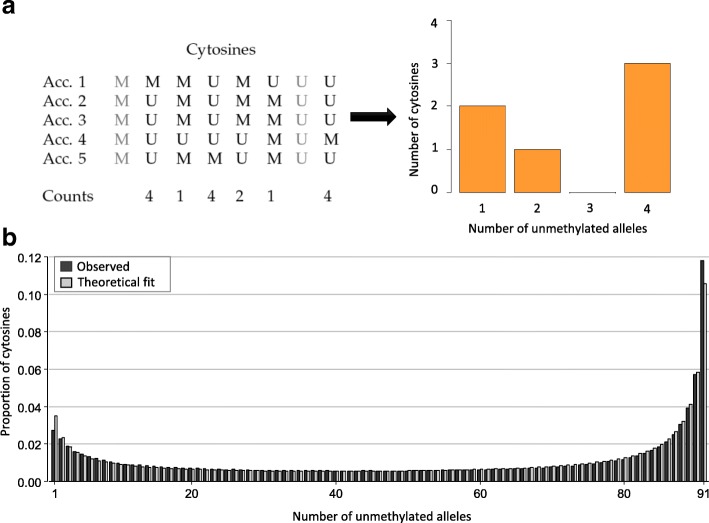
**a** Simplification of the reconstruction of a methylation site frequency spectrum (mSFS). In this example, we consider a sample size of five accessions (Acc.), and eight sites among which two (in *gray*) are monomorphic and thus discarded for the mSFS. For each cytosine, each accession might exhibit a methylated (M) or an unmethylated (U) state. For the mSFS, counts are taken of the number of accessions that are unmethylated for that cytosine. These counts define discrete epiallelic classes (number of unmethylated alleles). **b** The observed frequencies of each epiallelic class is determined, in this case, from genic CG sites of 92 A. thaliana worldwide natural accessions (*red bars*), along with the maximum likelihood estimate based on the theoretical result of Charlesworth and Jain [123] (*pink bars*). The theoretical model (see Box 1) provides an accurate fit to the observed genic CG methylation diversity patterns, suggesting that CG epimutations are a major factor in shaping methylome diversity in natural populations of A. thaliana over evolutionary timescales
