# Supplementary material for: Methylome evolution in plants
Source: Genome Biol. 2016 Dec 20;17:264. doi: 10.1186/s13059-016-1127-5 (PMC5175322; doi:10.1186/s13059-016-1127-5)
Supplement: Additional file 1: — Plant species whose methylomes have been analyzed by whole-genome bisulfite sequencing (WGBS-seq) or by high-performance liquid chromatography (HPLC). (PDF 277 kb) [file 13059_2016_1127_MOESM1_ESM.pdf]

**Additional file 1**

Plant species whose methylomes have been analyzed by whole-genome bisulfite sequencing (WGBS-seq) or high-performance liquid chromatography (HPLC)

| Species | Family | WGBS-seq | HPLC |
|---|---|---|---|
| *Amborella trichopoda* | *Amborellaceae* | Genome | |
| *Aquilegia nevadensis* | *Ranunculaceae* | | X |
| *Aquilegia vulgaris* | *Ranunculaceae* | | X |
| *Arabidopsis lyrata* | *Brassicaceae* | Genome | |
| *Arabidopsis thaliana* | *Brassicaceae* | Genome | X |
| *Aristolochia fimbriata* | *Aristolochiaceae* | EST | |
| *Asplenium nidus* | *Aspleniaceae* | RNA-seq | |
| *Beta vulgaris* | *Chenopodiaceae* | Genome | |
| *Brachypodium distachyon* | *Poaceae* | Genome | |
| *Brassica napus* | *Brassicaceae* | | X |
| *Brassica oleracea* | *Brassicaceae* | Genome | X |
| *Brassica rapa* | *Brassicaceae* | Genome | |
| *Cannabis sativa* | *Cannabaceae* | Genome | |
| *Capsella rubella* | *Brassicaceae* | Genome | |
| *Cichorium intybus* | *Asteraceae* | | X |
| *Citrus clementina* | *Rutaceae* | Genome | |
| *Crocus sativus* | *Iridaceae* | Genome | |
| *Cycas rumphii* | *Cycadaceae* | EST | |
| *Cymbidium pumilum* | *Orchidaceae* | | X |
| *Daphne blagayana* | *Thymelaeaceae* | | X |
| *Daphne laureola* | *Thymelaeaceae* | | X |
| *Daphne mezereum* | *Thymelaeaceae* | | X |
| *Echinochloa frumentacea* | *Poaceae* | | X |
| *Elaeis guineensis* | *Arecaceae* | | X |
| *Eleusine coracana* | *Poaceae* | | X |
| *Erodium cazorlanum* | *Geraniaceae* | | X |
| *Eucalyptus grandis* | *Myrtaceae* | Genome | |
| *Eutrema salsugineum* | *Brassicaceae* | Genome | |
| *Fragaria vesca* | *Rosaceae* | Genome | |
| *Gentiana pannonica* | *Gentianaceae* | | X |
| *Glycine max* | *Fabaceae* | Genome | |
| *Gossypium hirsutum* | *Malvaceae* | | X |
| *Gossypium raimondii* | *Malvaceae* | Genome | |
| *Helianthus annuus* | *Asteraceae* | | X |
| *Helleborus foetidus* | *Ranunculaceae* | | X |
| *Helleborus lividus* | *Ranunculaceae* | | X |
| *Ilex aquifolium* | *Aquifoliaceae* | | X |
| *Lavandula latifolia* | *Lamiaceae* | | X |
| *Lepidium sativum* | *Brassicaceae* | | X |
| *Linum usitatissimum* | *Linaceae* | | X |
| *Liriodendron tulipifera* | *Magnoliaceae* | EST | |
| *Lobularia maritima* | *Brassicaceae* | | X |
| *Lotus japonicus* | *Fabaceae* | Genome | |
| *Malus domestica* | *Rosaceae* | Genome | |
| *Manihot esculenta* | *Euphorbiaceae* | Genome | |
| *Marchantia polymorpha* | *Marchantiaceae* | RNA-seq | |
| *Medicago truncatula* | *Fabaceae* | Genome | |
| *Metroxylon sagu* | *Arecaceae* | | X |
| *Mimulus guttatus* | *Phrymaceae* | Genome | |
| *Myrtus communis* | *Myrtaceae* | | X |
| *Narcissus bugei* | *Amaryllidaceae* | | X |
| *Narcissus longispathus* | *Amaryllidaceae* | | X |
| *Narcissus nevadensis* | *Amaryllidaceae* | | X |
| *Nicotiana tabacum* | *Solanaceae* | | X |
| *Nuphar advena* | *Nymphaeaceae* | EST | |
| *Olea europaea* | *Oleaceae* | | X |
| *Oryza sativa* | *Poaceae* | Genome | X |
| *Panicum hallii* | *Poaceae* | Genome | |
| *Panicum virgatum* | *Poaceae* | Genome | X |
| *Pennisetum glaucum* | *Poaceae* | | X |
| *Phillyrea latifolia* | *Oleaceae* | | X |
| *Physcomitrella patens* | *Funariaceae* | Genome | |
| *Picea glauca* | *Pinaceae* | RNA-seq | |
| *Pinguicula vulgaris* | *Lentibulariaceae* | Genome | |
| *Pinus taeda* | *Pinaceae* | Genome | |
| *Pisum sativum* | *Fabaceae* | | X |
| *Populus trichocarpa* | *Salicaceae* | Genome | |
| *Primula vulgaris* | *Primulaceae* | | X |
| *Prunus persica* | *Rosaceae* | Genome | |
| *Pteridium aquilinum* | *Dennstaedtiaceae* | RNA-seq | |
| *Pyrus communis* | *Rosaceae* | | X |
| *Quercus ilex* | *Fagaceae* | | X |
| *Quercus suber* | *Fagaceae* | | X |
| *Rhinanthus minor* | *Orobanchaceae* | | X |
| *Ricinus communis* | *Euphorbiaceae* | Genome | |
| *Rosmarinus officinalis* | *Lamiaceae* | | X |
| *Secale cereale* | *Poaceae* | | X |
| *Selaginella moellendorffii* | *Selaginellaceae* | Genome | |
| *Setaria italica* | *Poaceae* | | X |
| *Setaria viridis* | *Poaceae* | Genome | |
| *Sinapis alba* | *Brassicaceae* | | X |
| *Solanum lycopersicum* | *Solanaceae* | Genome | X |
| *Solanum tuberosum* | *Solanaceae* | | X |
| *Sorghum bicolor* | *Poaceae* | Genome | X |
| *Stellaria longipes* | *Caryophyllaceae* | | X |
| *Theobroma cacao* | *Malvaceae* | Genome | |
| *Triticum aestivum* | *Poaceae* | | X |
| *Vicia faba* | *Fabaceae* | | X |
| *Viola cazorlensis* | *Violaceae* | | X |
| *Viscum album* | *Viscaceae* | | X |
| *Vitis vinifera* | *Vitaceae* | Genome | |
| *Zea mays* | *Poaceae* | Genome | X |

Abbreviation: EST, expressed sequence tags
