# Supplementary material for: Methylome evolution in plants
Source: Genome Biol. 2016 Dec 20;17:264. doi: 10.1186/s13059-016-1127-5 (PMC5175322; doi:10.1186/s13059-016-1127-5)
Supplement: Additional file 2: Figure S1. — GMLs of different taxa measured by HPLC and WGBS-seq. Figure S2. Correlation between genome size and total number of repeats in the genome. (PDF 1044 kb) [file 13059_2016_1127_MOESM2_ESM.pdf]

Figure S1

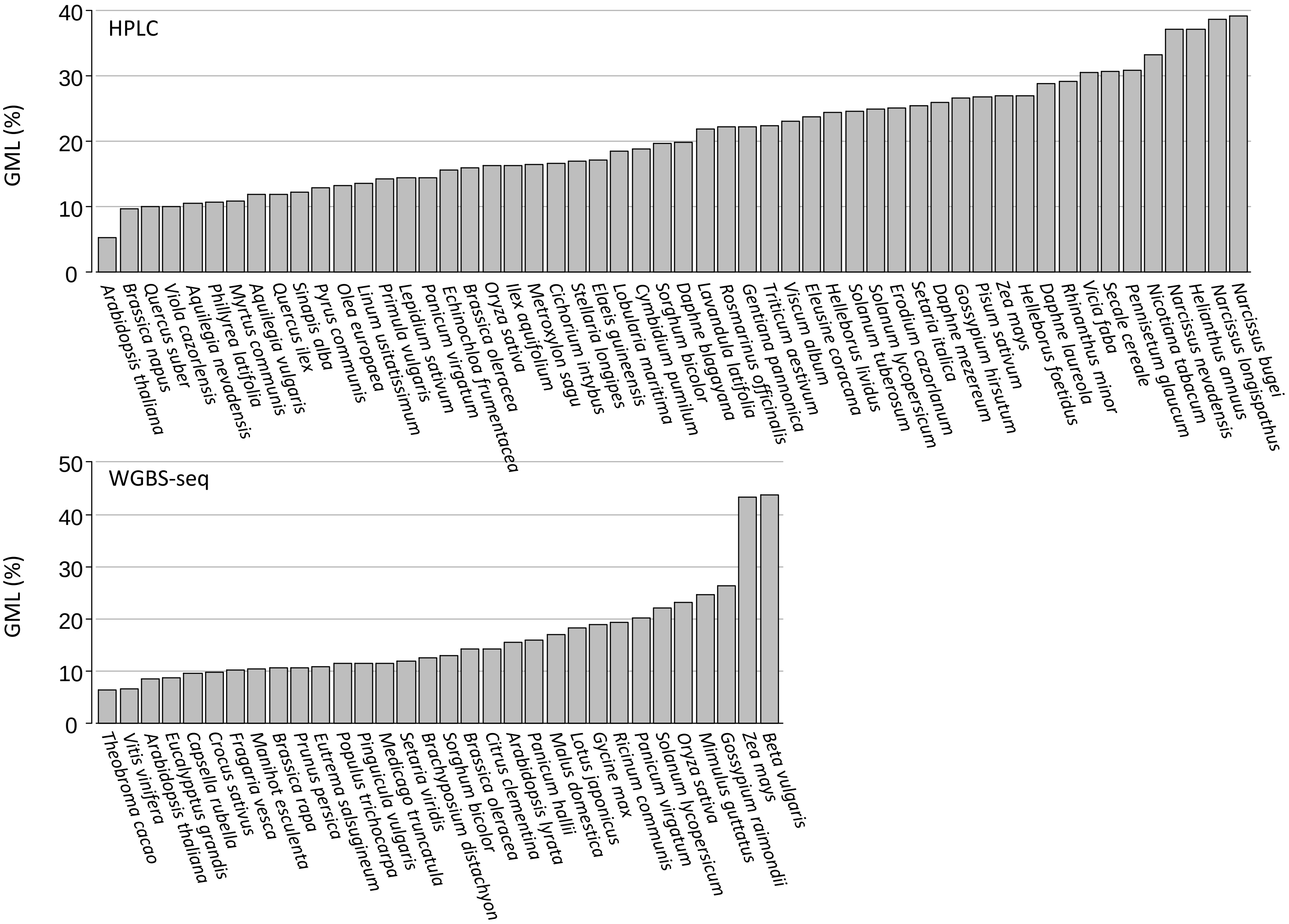

Figure S1: GMLs of plant species whose methylomes have been analyzed by whole genome bisulfite sequencing (WGBS-seq) or high-performance liquid chromatography (HPLC)

Figure S2

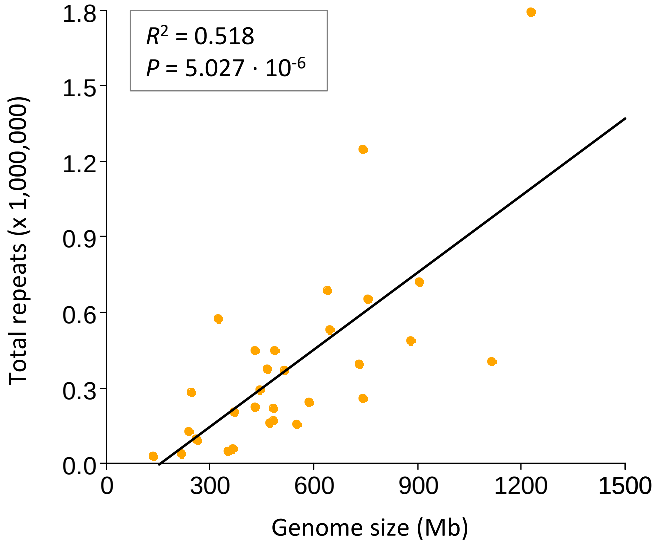

Figure S2: Correlation between genome size and total number of repeats in the genome, using the taxa presented in S1
