# Supplementary material for: Methylome evolution in plants
Source: Genome Biol. 2016 Dec 20;17:264. doi: 10.1186/s13059-016-1127-5 (PMC5175322; doi:10.1186/s13059-016-1127-5)
Supplement: Additional file 3: — Filtering of the methylomes used for the calculation of the mSFS. (DOCX 17 kb) [file 13059_2016_1127_MOESM3_ESM.docx]

# Additional file 3

# Supplementary Information

# Methylome evolution in plants

Amaryllis Vidalis^1†,^, Daniel Živković^2†,^, René Wardenaar^3^, David Roquis^1^, Aurélien Tellier^2*^, Frank Johannes^1*^

^†^Contributed equally

^1^Population Epigenetics and Epigenomics, Technical University of Munich, Liesel-Beckman-Str. 2, 85354 Freising Germany

^2^Population Genetics, Technical University of Munich, Liesel-Beckman-Str. 2, 85354 Freising Germany

^3^Groningen Bioinformatics Centre, University of Groningen, 9747 AG Groningen, The Netherlands

^*^Correspondence: tellier@wzw.tum.de; frank@johanneslab@org

# Filtering of the methylomes used for the calculation of the mSFS

For the computation of the methylation site frequency spectrum (mSFS) on the gene body single methylation polymorphisms (SMPs) (for the unmethylated allele, Fig. 5), we filtered the available methylomes as follows.

The methylomes were downloaded from NCBI GEO (GSE43857) [1]. Datasets from 138 accessions that comprised both SNP and SMP information were included in the analysis.

The methylome data we used were restricted to the reference CG positions for among-accessions comparisons. Methylome data were available from bud and/or from leaf tissue. For the accessions that had methylomes from both tissues, we selected the datasets with the highest number of reference CGs with sufficient coverage (>4). Five percent (7) of the accessions with the lowest number of reference CGs (with sufficient coverage) were excluded, resulting in 131 accessions, including the reference Col_0. After the filtering at the accessions level, we performed an additional filtering at the CG-positions level: we included only the CG positions for which at least 70 % (92) of the accessions had sufficient coverage and were polymorphic (methylated allele >0 and unmethylated allele >0). As the calculation of a SFS cannot handle missing data, for the CG positions with missing data, we imputed from the existing data with random resampling. This resulted in the final 92 accessions and in 457,518 DMPs (representing the Gene Body annotation category) used for the SFS that was calculated to fit the evolutionary model [2] (Fig. 5).

# References

1. Schmitz RJ, Schultz MD, Urich MA, Nery JR, Pelizzola M, Libiger O, et al. Patterns of population epigenomic diversity. Nature. 2013;495:193–8.

2. Charlesworth B, Jain K. Purifying selection, drift, and reversible mutation with arbitrarily high mutation rates. Genetics. 2014;198:1587–602.
